# Case report: orthodromic atrioventricular re-entrant tachycardia with left lateral accessory pathway—smart diagnosis with a smart device

**DOI:** 10.1093/ehjcr/ytaf177

**Published:** 2025-04-08

**Authors:** Caroline M Wiederkehr, Andreas S Müller, Martin Igual, Alain M Bernheim

**Affiliations:** Department of Cardiology, Stadtspital Zürich Triemli, Birmensdorferstrasse 497, 8063 Zürich, Switzerland; Department of Cardiology, Stadtspital Zürich Triemli, Birmensdorferstrasse 497, 8063 Zürich, Switzerland; Herzpraxis Zürich Nord, Nansenstrasse 16, 8050 Zürich, Switzerland; Department of Cardiology, Stadtspital Zürich Triemli, Birmensdorferstrasse 497, 8063 Zürich, Switzerland

**Keywords:** Case report, Arrhythmia, ECG, Wearable smart device, Electrophysiology

## Abstract

**Background:**

Patients with supraventricular tachycardia (SVT) are often symptomatic, but frequency of symptoms is very variable. Definition of tachycardia mechanism and diagnosis is dependent on documentation of the arrhythmia by electrocardiogram (ECG), but this documentation is often missed by standard evaluation with a Holter ECG. Wearable smart devices with ECG function are valuable diagnostic tools in such patients.

**Case summary:**

We describe a case of a 59-year-old male patient who suffered from infrequent palpitations, where documentation of two distinct tachycardia ECG tracings with a wearable smart device led to the correct diagnosis. One tracing showed an episode of narrow QRS complex tachycardia at a heart rate of 200 b.p.m. and the other a broad complex tachycardia with left bundle branch block morphology at a slower heart rate. Based on these findings, which demonstrate Coumel’s sign, atrioventricular re-entrant tachycardia with left lateral accessory pathway was suspected. Electrophysiological study confirmed the diagnosis, and an accessory pathway located at the lateral mitral isthmus was successfully ablated.

**Discussion:**

Aberrant ventricular conduction due to functional bundle branch block is an important finding during SVT. In the presented case, a wearable smart device was able to document two episodes of tachycardia with two distinct ECG morphologies, one with broad QRS complexes at a slower heart rate and the other with a faster episode of narrow QRS complexes. Therefore, the wearable device was not only able to document an episode of symptomatic tachycardia, but it also additionally offered important keys to the correct diagnosis.

**Learning points**

- Smart wearable devices with electrocardiogram function offer a convenient and easy-to-use diagnostic modality for longer rhythm diagnostics in a selected patient group who complain about paroxysmal palpitations.
- Slowing of supraventricular tachycardia during episodes of functional bundle branch block is diagnostic for orthodromic atrioventricular re-entrant tachycardia with an accessory pathway at the side of the bundle branch block (‘Coumel’s sign’).

## Introduction

Wearable smart devices are increasingly used as a diagnostic tool in patients with palpitations.^1^ These devices are equipped with photoplethysmography and single-lead electrocardiogram (ECG) recording technologies and are available as a direct-to-consumer product at a relatively low cost.^2,3^ Therefore, they are an accessible, convenient, and easy-to-use tool that may help to detect paroxysmal arrhythmias as the underlying cause of patients’ symptoms.

To date, there is inconsistent quality control and lack of validation for accuracy for many of the available wearable smart devices.^3–5^ However, their diagnostic value can be enhanced by manual review and verification of recorded rhythm strips by a trained professional.^6^

We describe a patient who recorded ECG tracings with a wearable smart device during an ongoing episode of palpitations, where diagnosis of atrioventricular re-entrant tachycardia (AVRT) with left lateral pathway was suspected based on specific ECG characteristics. The patient was scheduled for an electrophysiological (EP) study, where the accessory pathway (AP) was localized and successfully ablated at the lateral mitral annulus.

## Summary figure

**Figure:**
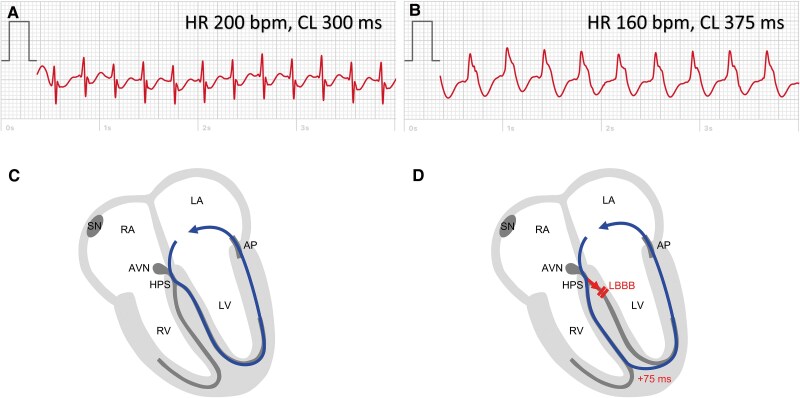


## Case presentation

A 59-year-old man presented to a cardiologist outpatient clinic because of palpitations. He reported a 1-year history of recurrent palpitations. He suffered from episodes of regular tachycardia with sudden onset, lasting for ∼5 min. These episodes increased in frequency over the last few weeks.

**Figure 1:**
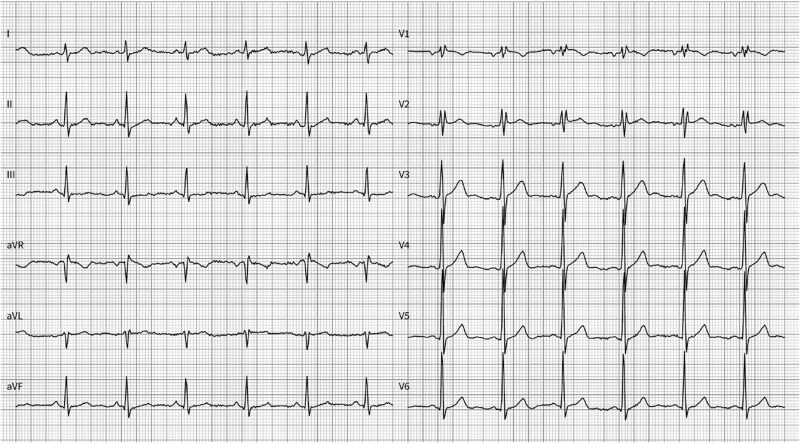
Baseline electrocardiogram with incomplete right bundle branch block, normal timing intervals (PQ 160 ms, QRS 107 ms, QTc 441 ms, and heart rate 75 b.p.m.), and no ventricular pre-excitation.

Medical history was unremarkable and the patient took no medication. He was free from other cardiac symptoms and reported no limitation in his exercise capacity. Cardiovascular risk factors were absent. Clinical findings were unremarkable with normal blood pressure and heart rate (HR) measurements, normal weight and body mass index, and otherwise normal physical examination. Baseline ECG showed sinus rhythm (SR) (HR 75 b.p.m.), normal PQ interval (160 ms) without signs of ventricular pre-excitation, QRS width of 107 ms with incomplete right bundle branch block (BBB), and normal repolarization including QTc interval of 441 ms (*Figure 1*).

Transthoracic echocardiography showed no signs of structural heart disease, with normal size and function of both ventricles, normal size of both atria, and absence of relevant valvulopathies. Stress echocardiography on a treadmill revealed good exercise capacity, the patient was asymptomatic during stress testing, ECG was without signs of ischemia, and no wall motion abnormalities were detected during exercise. A 48-h Holter ECG showed one non-sustained atrial tachycardia with 1:1 conduction to the ventricle at a HR of 147/min (11 narrow QRS complexes); the patient reported no correlating palpitations or other symptoms during the recording.

Because of lacking ECG documentation of the clinical tachycardia, diagnosis could not be made. In the absence of signs for malignant arrhythmia or atrial fibrillation, a supraventricular re-entrant tachycardia was suspected, and the patient was instructed in vagal manoeuvres.

**Figure 2:**
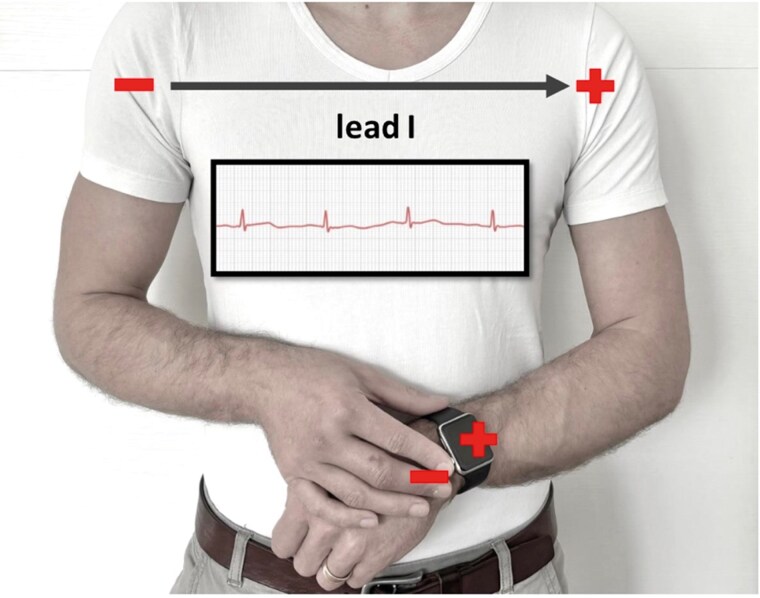
Recording of an electrocardiogram with a smart device (in this example apple watch). The circuit is closed by touching the index finger of the right hand to the crown of the watch (= anode; cathode at the back of the watch), which is worn on the left wrist, thereby recording Lead I.

The patient was informed about the option of wearable smart devices to document a single-lead ECG during another episode of palpitations. With these devices, ECG tracings are usually recorded while touching the crown of the watch at the left wrist by the right index finger, so the traced lead corresponds to Lead I (*Figure 2*).

**Figure 3:**
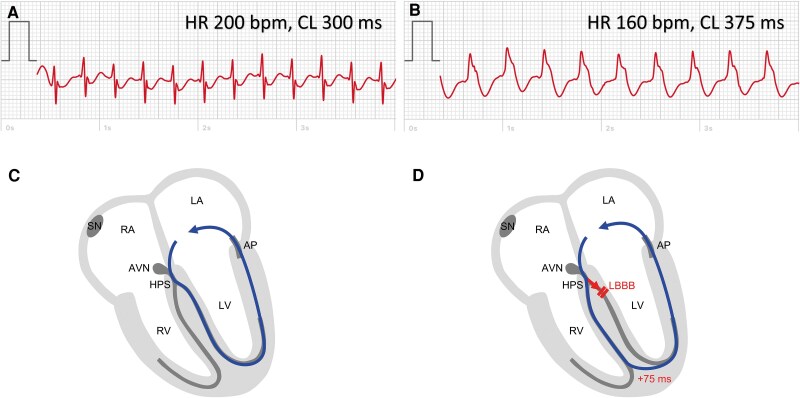
Electrocardiogram tracings and underlying mechanisms during narrow complex and wide complex orthodromic atrioventricular re-entrant tachycardia. (*A*) Narrow complex tachycardia, heart rate 200 b.p.m., cycle length 300 ms, corresponding to *C*, showing normal conduction through left and right bundle branch and retrograde conduction through a left lateral accessory pathway. (*B*) Broad complex tachycardia, heart rate 160 b.p.m., cycle length 375 ms, corresponding to *D*, showing functional left bundle branch block during tachycardia and consecutive conduction delay. AVN, atrioventricular node; AP, accessory pathway; HPS, His-Purkinje system; LA, left atrium; LV, left ventricle; RA, right atrium; RV, right ventricle; SN, sinus node.

The patient continually suffered from episodic symptoms, and documentation of a corresponding tachycardia with a smart device was successful. The ECG recordings of various episodes demonstrated a regular wide QRS complex tachycardia (HR 145–160 b.p.m.) with a left BBB (LBBB) morphology (broad, positive, and notched QRS complexes). One day, during an ongoing episode of palpitations, the patient traced one ECG with a wide LBBB QRS complex tachycardia [HR 160 b.p.m. and cycle length (CL) 375 ms] and another with a narrow QRS complex tachycardia (HR 200 b.p.m. and CL 300 ms) (*Figure 3*). Coumel *et al*.^7^ first described the prolongation of the tachycardia CL in combination with functional BBB. This sign is consistent with an orthodromic AVRT using an AP located at the same side of the heart as the functional BBB (*Figure 3*).

The patient was referred for invasive EP study. During the procedure, no ventricular pre-excitation was noted during atrial pacing. Eccentric retrograde conduction suggestive for an AP was evident during ventricular pacing. Programmed ventricular stimulation triggered the symptomatic tachycardia with a CL of 380 ms, LBBB morphology, and eccentric retrograde activation of the left atrium. No other arrhythmia was induced during the EP study.

**Figure 4:**
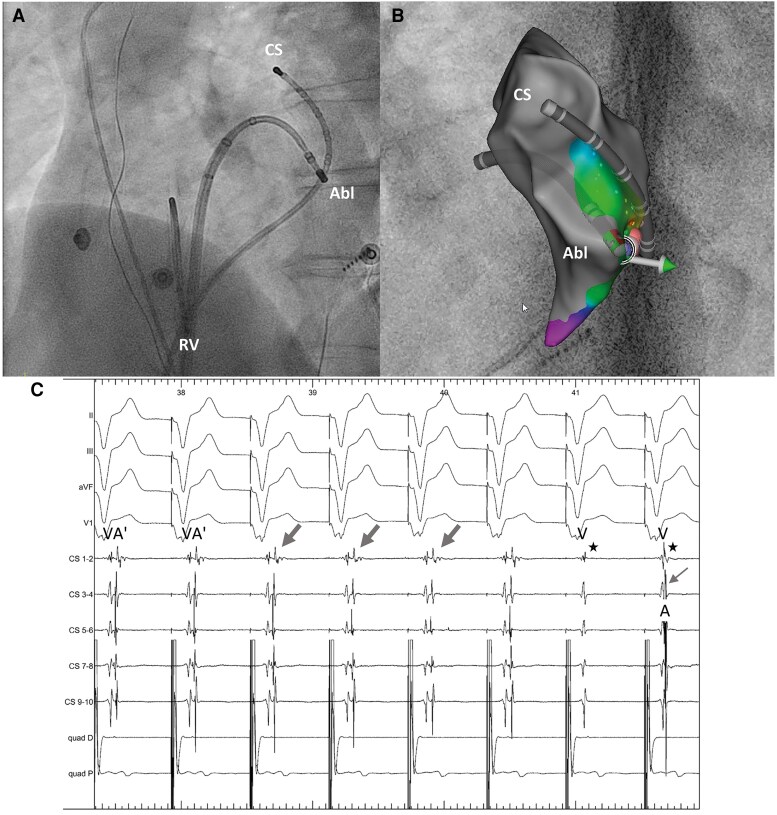
Ablation setting and block of retrograde conduction during ablation on lateral mitral annulus. (*A*) Left anterior oblique view of the heart; quadripolar catheter in the right ventricle, decapolar catheter in coronary sinus, and ablation catheter on the lateral mitral annulus (transseptal approach). (*B*) Enlarged view of fluoroscopic view (*A*) and 3D map (Carto, UniVue) of lateral mitral annulus region with catheter projections. (*C*) Electrocardiogram and electrograms during ablation on lateral mitral annulus during pacing from quadripolar catheter in the right ventricle; double signal (broad arrow) on coronary sinus catheter electrograms, first spike corresponding to far-field sensing of ventricular depolarization (V), second spike corresponding to local retrograde atrial (A′) depolarization (first 6 beats); block of retrograde conduction (evident in the last 2 beats, no more second signal, only far-field ventricular signal on coronary sinus catheter, asterisk), intrinsic atrial depolarization (A) via sinus node on last beat (narrow arrow) achieved after 4.8 s of ablation. Abl, ablation catheter; CS, coronary sinus; RV, right ventricle.

The AP was localized at the lateral mitral annulus and successfully ablated using a transseptal approach and a 3D mapping system (*Figure 4*).

## Discussion

Our case illustrates the diagnostic value of wearable smart devices in a case of paroxysmal supraventricular tachycardia (SVT). Together with the ECG in SR, the recordings demonstrated four important findings suggesting the underlying mechanism: (i) normal ECG without pre-excitation or BBB in SR; (ii) ongoing regular tachycardia with narrow and wide QRS complexes, (iii) wide QRS complexes with LBBB morphology, and (iv) two different HR.

**Figure 5:**
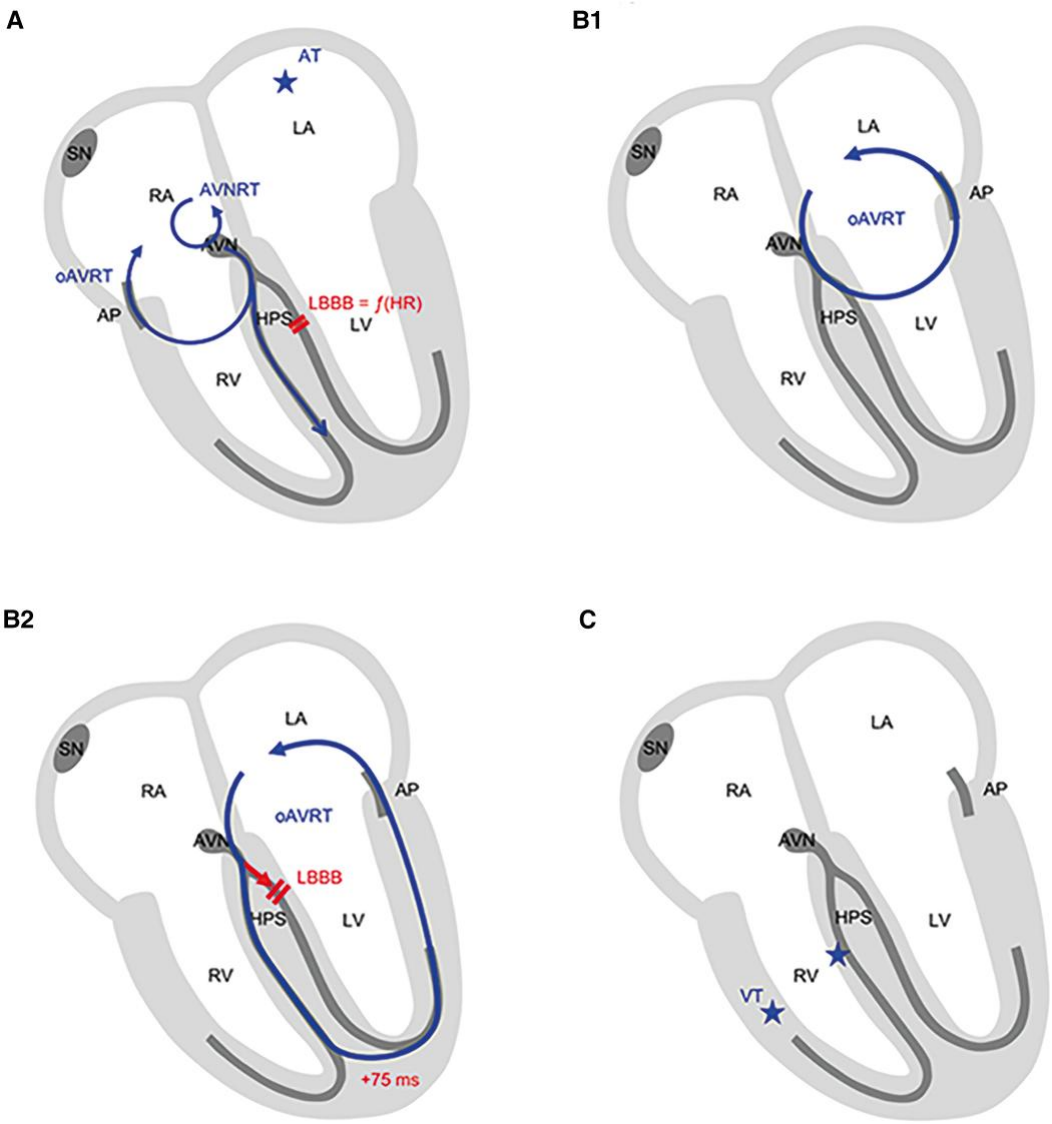
(*A*) Atrial tachycardia, atrioventricular nodal re-entrant tachycardia, or orthodromic atrioventricular re-entrant tachycardia (right/septal accessory pathway) with functional left bundle branch block. (*B1*) Orthodromic atrioventricular re-entrant tachycardia (left accessory pathway) with normal conduction and without functional left bundle branch block. (*B2*) Orthodromic atrioventricular re-entrant tachycardia (left accessory pathway) with functional left bundle branch block. (*C*) Right-sided ventricular tachycardia. AP, accessory pathway; AT, atrial tachycardia; AVNRT, atrioventricular nodal re-entrant tachycardia; LBBB, left bundle branch block; oAVRT, orthodromic atrioventricular re-entrant tachycardia; VT, ventricular tachycardia.

Supraventricular tachycardia typically presents as narrow complex tachycardias, but pre-existing or functional BBB can lead to wide complex tachycardia during SVT. Potential differential diagnoses of the tachycardia found in our patient are presented in *Table 1* and *Figure 5*. The comparison of the HRs during tachycardia with and without aberrant ventricular conduction provides an important clue to the correct diagnosis. Heart rates during SVT can vary depending on adrenergic tone. If the presented case of LBBB wide complex tachycardia was an atrial tachycardia or an atrioventricular nodal re-entrant tachycardia with aberrancy, wide complexes would rather occur at higher HRs, and not during episodes of lower HR, as in our case (*Figure 5A* and *Panel A* in *Table 1*).

**Table 1:** Tachycardia mechanism electrocardiogram features

Panel	Mechanism	ECG features	QRS in tachycardia	TCL (narrow vs. wide)	LBBB morphology
		QRS in sinus rhythm			
A	AT, AVNRT, or oAVRT (r/s cAP) with functional LBBB	Narrow	Narrow and wide	Usually TCL (narrow) > TCL (wide)	Rate dependent
B	oAVRT (left cAP) with functional LBBB	Narrow	Narrow and wide	TCL (narrow) < TCL (wide)	LBBB at longer TCL
C	VT (r/s)	Narrow	Only wide	No effect on QRS morphology	Only in tachycardia

AT, atrial tachycardia; AVNRT, atrioventricular nodal re-entrant tachycardia; LBBB, left bundle branch block; oAVRT, orthodromic atrioventricular re-entrant tachycardia; TCL, tachycardia cycle length; r/s cAP, right-sided or septal concealed accessory pathway; VT, ventricular tachycardia.

Antidromic AVRT (conduction of tachycardia antegradely over an AP and retrogradely via the AV node) is also a possible mechanism of wide complex SVT. Wide complex tachycardia with LBBB morphology, such as presented in our case, would correspond to antidromic AVRT with right-sided pathway. However, antidromic AVRT was not considered very likely, since there was no evidence for ventricular pre-excitation on the ECG. With an overt AP, both orthodromic and antidromic AVRTs could occur, but it is not possible for both forms of AVRT to appear in the same uninterrupted episode of tachycardia.

The absence of ventricular pre-excitation in SR does not exclude AVRT as pathomechanism of SVT, because only retrogradely conducting AP, so-called concealed pathway, could be present. Orthodromic AVRT (antegrade conduction over the AV node and retrograde conduction via AP) typically presents as narrow complex tachycardia (*Figure 5B1*). Functional BBB can occur during orthodromic AVRT, then presenting as wide complex tachycardia (*Panel B* in *Table 1*; *Figure 5B2*). Also in this case, HRs of the different tachycardia episodes should be compared. Orthodromic AVRT via a right lateral pathway (e.g. at the contralateral side of the BBB) does not slow down during episodes of LBBB.^8^ The prolongation of the CL with a slower HR during aberrancy is characteristic of orthodromic AVRT involving an AP located on the side of the BBB. This phenomenon, attributed to the longer ventricular distance and extended conduction time through working myocardium compared with the His-Purkinje system, was first described by P. Coumel in 1974.^1^

All the above considerations led to the working diagnosis of orthodromic AVRT with left-sided pathway, which could be confirmed in an EP study.

Epidemiological data on SVT patients are limited. Prevalence of non-atrial fibrillation SVT is 2.25/1000 persons with a female predilection.^9–11^ Patients are often symptomatic, but frequency of symptoms is very variable. Definition of tachycardia mechanism and diagnosis is dependent on documentation of the arrhythmia with ECG, with higher rates of detection during longer observation periods.^12,13^ Smart devices are a fast-evolving innovation in the field of diagnostic tools, with increasing accuracy in measurement of HR and improvement in ECG quality during SR and tachycardia^14^ Nowadays, smart devices are widely used and dedicated devices are suitable for documentation of ECG tracings during an episode of palpitations. By photoplethysmography, HR and pulse regularity can be assessed, and through single-lead ECGs, HR and QRS morphology can be recorded. This may facilitate detection of rhythm disturbances that had not been diagnosed by conventional rhythmological examinations, such as Holter ECG. However, sensitivity and specificity vary among different smart devices, and manual review of the device detected arrhythmia is still mandatory.^6^ In our case, the evaluation of the morphology and width (narrow vs. wide) of the QRS complexes in the ECG tracings were made visually and on the assumption that the device was worn on the left wrist. These factors clearly impact accuracy and the evaluation of such tracings, especially compared with traditional ECG documentation (e.g. Holter ECG), where analysis and exact measurements can be made in a standardized manner. Further limitations of smart devices are multifaceted: the lacking regulatory standards and validation processes, privacy and safety of this so-called patient-generated health data (PGHD), absence of structured PGHD management or infrastructure, possible behavioural and psychological impact, and adherence to or engagement with the wearables to name a few.^4^ Moreover, body temperature, skin tone, hair, and tattoos as well as movement may impact data quality.^15^ Unequal access to and use of digital health technologies, either for economical, geographical, or social reasons, is another limitation. According to a study conducted in the USA, there is a substantial underuse of smart devices among patients with cardiovascular disease, who could possibly gain the most from the use of innovative technologies for disease management.^16–18^

Despite these limitations, smart wearable devices offer a convenient and easy-to-use new diagnostic modality for longer rhythm diagnostics in a selected patient group who complain about paroxysmal palpitations. In our case, the smart wearable device led to successful ECG documentation of symptomatic tachycardia and provided important clues to the underlying arrhythmia mechanism.

## Data Availability

Data used in this case report are available to readers upon request.

## Lead author biography

Dr Caroline M. Wiederkehr is currently an EP fellow at Stadtspital Zurich Triemli, Switzerland. She completed her Master of Medicine at the University of Zurich, Switzerland, in 2015 and has trained as a resident in anaesthesiology, internal medicine, and cardiology.

## Notes

**Consent:** Informed written patient consent has been obtained in line with the COPE best practice guidelines.

**Funding:** This case report was not supported by any funding.
